# Influence of red mud and waste glass on the microstructure, strength, and leaching behavior of bottom ash-based geopolymer composites

**DOI:** 10.1038/s41598-020-76818-4

**Published:** 2020-11-13

**Authors:** Constantin Bobirică, Cristina Orbeci, Liliana Bobirică, Petru Palade, Călin Deleanu, Cristian Mircea Pantilimon, Cristian Pîrvu, Ionuţ Cristian Radu

**Affiliations:** 1 Department of Analytical Chemistry and Environmental Engineering, University Politehnica of Bucharest, 1-7 Polizu, 011061 Bucharest, Romania; 2 National Institute of Materials Physics, P.O. Box MG-7, 077125 Bucharest-Magurele, Romania; 3 “C.D. Neniţescu”, Center of Organic Chemistry, 202-B Spl. Independenţei, 060023 Bucharest, Romania; 4 Department of Metallic Materials Processing and Eco-Metallurgy, University Politehnica of Bucharest, 313 Spl. Independenţei, 060042 Bucharest, Romania; 5 Department of General Chemistry, University Politehnica of Bucharest, 1-7 Polizu, 011061 Bucharest, Romania; 6 Department of Bioresources and Polymer Science, University Politehnica of Bucharest, 1-7 Polizu, 011061 Bucharest, Romania

**Keywords:** Environmental sciences, Chemistry, Engineering, Nanoscience and technology

## Abstract

The influence of waste glass and red mud addition as alternative source of aluminosilicate precursors on the microstructural, mechanical, and leaching properties of bottom ash-based geopolymer was studied in this work through mineralogical, morphological, and spectroscopic analysis, as well as by conducting compressive strength and leaching tests. The bottom ash-based geopolymer composites were synthesized by adding a constant amount of waste glass (10% by weight) and increasing amounts of red mud (up to 30% by weight). The results derived from FTIR, ^29^Si and ^27^Al MAS NMR, and SEM–EDX revealed that adding up to 10% (by weight) red mud to the synthesis mixes leads to an increase in the degree of geopolymerization of the activated mixes. The compressive strength followed the same trend. An increase of more than 10% (by weight) red mud added to the synthesis mixes results in a significant decrease of compressive strength of the geopolymer composites. A low leachability of geopolymer composites in regard with their contaminants was revealed especially for those with good compressive strength.

## Introduction

Nowadays there is an increasing interest worldwide to enhancing the valorization of secondary raw materials in order to preserve the primary raw materials and to reduce to a minimum the carbon footprint for a large range of products^1^. Building industry has a huge potential to valorize a wide category of industrial by-products and wastes to produce green and environment-friendly products so that to be a real alternative to conventional ones. Alkali-activated materials represent a promising category of materials in this regard^2^. Previous numerous studies investigated the opportunity to use a wide range of geopolymeric secondary raw materials to geopolymer synthesis such as fly ash, blast furnace slag, bottom ash, large assortments of waste glass, silica residue, red mud, rice husk ash, fayalite slag, etc.^3,4^.

From all of these geopolymeric precursors, bottom ash has received a lower attention due to its low reactivity mainly derived from the large and irregularly shape particles which are specific for this type of ash^5^. However, several authors have investigated the influence of alkali-activation conditions on the properties of bottom ash-based geopolymers. In this respect, Boca Santa et al.^6^ synthesized geopolymers using bottom ash as the sole source of aluminosilicates. Their results revealed that the geopolymers synthetized by using potassium hydroxide as activator and cured at room temperature have the best compressive strength. A comparative study regarding the properties of geopolymers synthetized from fly ash, and geopolymers synthetized from bottom ash revealed the similarity between them in terms of mechanical properties^5^. Topçu et al.^7^ found that for a good compressive strength the optimum Si/Al atomic ratio must be in the range of 3.5 to 4 and Si/Na atomic ratio must be close to 0.5. Studies regarding the influence of fly ash/bottom ash mass ratio on geopolymers properties highlight that an increase in this ratio leads to an increase in compressive strength of the synthetized geopolymers^8^. It was also found that geopolymers synthetized from bottom ash and paper sludge have a satisfactory geopolymerization degree and compressive strength^9^. Geopolymers synthetized from pre-treated bottom ash derived from the urban waste incinerator exhibit a porous morphology and an apparent density comparable to those of lightweight materials^10^. Geetha and Ramamurthy^11^ investigated the influence of calcium hydroxide addition on the properties of bottom ash-based geopolymers cured at ambient temperature, and they found that calcium hydroxide has a beneficial effect bringing the ambient temperature cured geopolymer properties in line with those of the oven-cured geopolymers. Adding of fly ash and waste gypsum in addition to bottom ash to the synthesis mixes leads to a significant increase in the strengths of bottom ash-based geopolymers^12^. Also, it was found that the fineness of bottom ash has a significant effect on the compressive strength of the obtained geopolymers, in the sense that an increase of the fineness leads to an increase of the compressive strength^13^.

Although the studies mentioned above highlight the possibility of using bottom ash to geopolymers synthesis with quite good results, additional research have to be carried out to establish the best synthesis conditions as a function of characteristics and reactivity of this type of ash. To the best of our knowledge, there are no studies regarding the use of waste glass or red mud in combination with bottom ash to geopolymer composites synthesis. Therefore, the aim of this work is to explore through mineralogical, microstructural, and spectroscopic analysis, as well as by conducting compressive strength and leaching tests, the influence of the addition of waste glass and red mud on the microstructure, strength, and leaching behavior of coal bottom ash-based geopolymer composites.

## Experimental section

### Materials and reagents

Coal bottom ash from a local coal-fired thermal power plant, waste glass derived from spent cathode-ray tubes (CRTs), and bauxite residue (red mud) from a local alumina refining industry, whose compositions are presented in Table 1, were used as raw geopolymeric precursors in this work. CRT glass cullet (glass from both panels and funnels of CRTs derived from spent TV sets and computer monitors) with dimensions in the range of 1 to 10 mm were provided by a local treatment plant for electric and electronic equipment waste (WEEE). The glass cullet was grinded up to a particles granulometry less than 75 μm into a ball mill. Both, coal bottom ash and red mud were oven dried and powdered to a particles granulometry less than 75 μm. A sodium hydroxide (NaOH, Sigma-Aldrich, analytical grade 98%, granulated) solution of 30% (percent by weight), which corresponds to a NaOH solution of 10 mol/L, was used as alkali activator. In all experiments, only deionized water was used.

**Table 1:** Chemical composition of the raw materials.

Concentration, % (by weight)			
Oxide	Bottom ash	Waste glass	Red mud
SiO_2_	47.40	49.60	11.00
Al_2_O_3_	24.80	5.70	21.00
CaO	3.30	1.50	5.63
Fe_2_O_3_	11.00	0.15	42.78
SO_3_	2.20	7.60	1.10
MgO	2.30	–	–
K_2_O	2.42	6.15	0.37
TiO_2_	0.87	–	2.37
Na_2_O	4.00	5.00	7.25
P_2_O_5_	–	0.20	7.85
Others	1.71	24.10	0.65

### Sample preparation

The geopolymer composites were prepared by mechanical mixing different mass ratios of bottom ash, waste glass, and red mud in the alkali activator solution (NaOH solution of 30% by weight) for 30 min, cast in polypropylene formworks (cylindrical shape, 5 cm × 10 cm), sealed off into plastic bags, and then cured for 24 h at 60 °C. Next, the samples are removed from formworks, sealed off into new plastic bags, and allowed to harden for the next 27 days at room temperature. Five different compositions were designed, for each composition being produced five replicates. The abbreviation used for the five compositions are as following: BA-N (bottom ash-sodium hydroxide solution); BA-WG-N (bottom ash-waste glass-sodium hydroxide solution); BA-WG-RM10-N (bottom ash-waste glass-red mud 10% by weight-sodium hydroxide solution); BA-WG-RM20-N (bottom ash-waste glass-red mud 20% by weight-sodium hydroxide solution); BA-WG-RM30-N (bottom ash-waste glass-red mud 30% by weight-sodium hydroxide solution). The mixes designing details are presented in Table 2.

**Table 2:** Composition of the synthesis mixes.

Component, % (by weight)					
Synthesis mixes	BA-N	BA-WG-N	BA-WG-RM10-N	BA-WG-RM20-N	BA-WG-RM30-N
Alkali activator	NaOH 30% (by weight)				
Bottom ash	66.67	56.67	46.67	36.67	26.67
Waste glass	–	10.00	10.00	10.00	10.00
Red mud	–	–	10.00	20.00	30.00
Na_2_O (activator)	7.75	7.75	7.75	7.75	7.75
H_2_O (activator)	25.58	25.58	25.58	25.58	25.58
Water/solid (w/s)	0.35	0.35	0.35	0.35	0.35
Na_2_O/solid (a/s)	0.12	0.12	0.12	0.12	0.12
**Molar ratios**					
Na_2_O/SiO_2_	0.37	0.35	0.20	0.14	0.10
Si_2_O/Al_2_O_3_	3.24	3.63	5.98	8.23	10.40
H_2_O/M_2_O	7.93	7.79	8.15	8.55	8.99
M_2_O/Al_2_O_3_	1.12	1.28	1.20	1.12	1.04

### Analysis and test methods

All the samples were oven dried and powdered to a particles granulometry less than 75 μm prior to be subjected to morphological, mineralogical and spectral analysis. It is worth mentioning that, the geopolymer composites subjected to such of analysis were further processed in order to stop the geopolymerization reaction. In this respect, the pulverized composite geopolymer samples were contacted for five minutes with a solvent consisting of a mixture of methanol and acetone (50/50 by volume), which then it was removed by vacuum filtration. The procedure was repeated three times. All the samples were kept in a desiccator under vacuum until to be used. The oxide composition of the geopolymeric precursors was determined by XRF analysis on a Philips PW 4025 MiniPal spectrophotometer. Mineralogical analysis was performed by using XRD diffractometry with a Panalytical X’Pert Pro MPD (Multipurpose Diffractometer). Data collection was done over a range from 10 to 90° with a scanning rate of 1.5° (2θ)/min with CuKα radiation (45 kV, 40 mA, λ = 1.5406 nm). The crystal phases were identified by referencing diffraction patterns in a licensed library from the International Centre for Diffraction Data (ICDD). A Bruker Vertex 70 FT-IR spectrophotometer with Attenuated Total Reflectance (ATR) accessory with 32 scans was use to record FT-IR spectra over a 4000—600 cm^−1^ range at a resolution of 4 cm^−1^. The ^29^Si and ^27^Al MAS-NMR spectra were recorded on a Bruker AVANCE III HD 600 MHz spectrometer (14.10 T, 4 mm ZrO_2_ rotors, ambient temperature, spinning rate 12 kHz). The ^29^Si spectra were acquired with a number of scans ranging between 1024 and 4096 scans depending on the sample, and a relaxation delay of 2 s. The ^27^Al spectra were acquired with a number of scans ranging between 128 and 4096, depending on the sample, and a relaxation delay of 1 s. The resonance frequency was 119.2 MHz for the Si nuclei and 156.4 MHz for the Al nuclei. The chemical shifts were measured relative to tetramethylsilane (TMS) as external reference for ^29^Si and Al(H_2_O)_6_^3+^ for ^27^Al. A Quanta 650 FEG scanning electron microscope (SEM) equipped with EDX analyzer operated at 10 kV was used to investigate the morphology of the samples. ^57^Fe Mössbauer spectroscopy in transmission geometry allowed us to obtain information concerning valence state, coordination, local distortion and relative amount of iron ions contained in various phases. The experiments were performed using an integrated system built from a SEECO spectrometer operating under constant acceleration mode and a ^57^Co(Rh) radioactive source. The Mössbauer spectra were fitted with NORMOS program^14^ which allows deconvolution of the experimental pattern in spectral components corresponding to different Fe non-equivalent positions. The leaching behavior of contaminants in the monolithic samples were investigated by carrying out monolithic leaching tests according to EPA Test Method 1315^15^. The test procedure consists of contacting (closed plastic bottles, without agitation, room temperature) the 28-day hardened monolithic samples (cylindrical shape, 5 cm × 10 cm) with deionized water at a liquid/solid ratio of 9 mL/cm^2^ of exposed surface area. The volume of leachant is renewed according to the following cumulative leaching time: 0.08, 1, 2, 7, 14, 28, 42, 49, and 63 days. The resulted leachates were analyzed to determine the concentration of the target contaminants by an Analytik-Jena spectrometer type contrAA-300. At the end of each leaching time, the leachants were filtered through 0.45 μm filter and then preserved with nitric acid to pH < 2 for chemical analyses of species of interest. The results were used to assess the mobility of contaminants in the monolithic samples by using their calculated leachability index (LI), which represents the negative logarithm of observed diffusivity (D_obs_, in unit of m^2^/s) of the contaminant in the monolithic sample (LI = pD_obs_ = − log D_obs_)^16^. Each leaching test was performed in duplicate. The compressive strength tests were carried out according to ASTM C39/C39M-14 standard procedure for cylindrical specimens^17^ on a Shimadzu CCM-200A testing machine. It is worth mentioning that, the 28-day hardened monolithic samples (cylindrical shape, 5 cm × 10 cm) were checked for their ends flatness, possible adjusted by sawing or grinding, and checked again for their dimensions prior to be subjected to compressive strength tests. Each compressive strength test was performed in triplicate.

## Results and discussion

### Compressive strength

**Figure 1:**
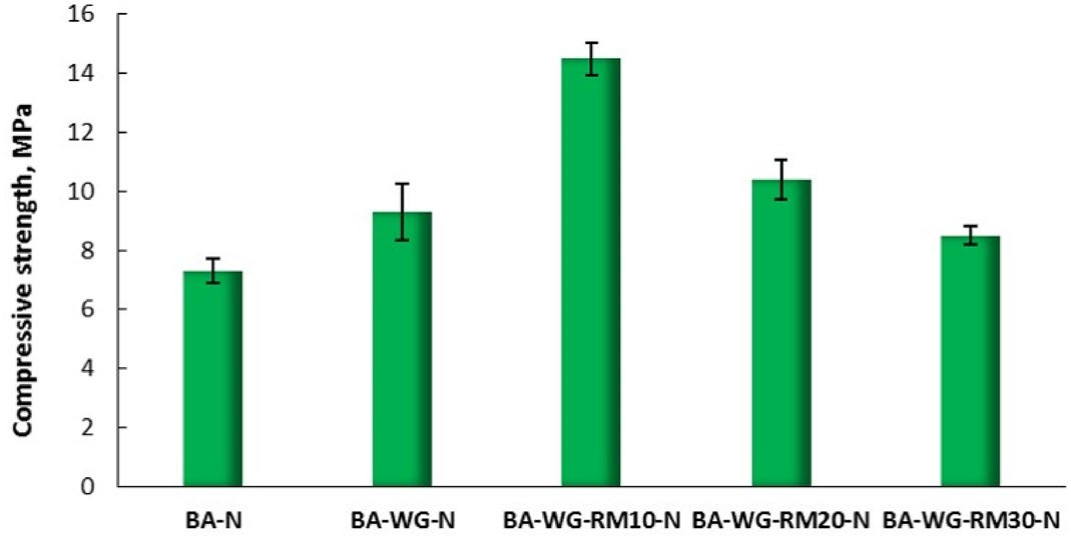
Compressive strength of the geopolymer composites. Error bars represent the calculated standard deviation for the experimental data.

The compressive strength of the geopolymer composites is shown in Fig. 1. As can be seen, there is an evident difference between the three types of geopolymer composites, the compressive strength increasing sharply in order BA-N (7.3 MPa) < BA-WG-N (9.3 MPa) < BA-WG-RM10-N (14.5 MPa). Also, it seems that a percentage of more than 10% red mud added to the synthesis mixes leads to a decrease of compressive strength of the geopolymer composites (BA-WG-RM10-N (14.5 MPa) > BA-WG-RM20-N (10.4 MPa) > BA-WG-RM30-N (8.5 MPa)). It is obvious that the addition of waste glass and red mud to the synthesis mixes has a strong impact on the composition of the geopolymer composites. Waste glass is an important source of dissolved silica in a strong alkaline environment, and therefore it is expected that its addition to the synthesis mixes will substantially alter the Si/Al molar ratio of the geopolymer composites. It is known that increasing the Si/Al molar ratio to a certain limit of which value depends on the synthesis conditions (i.e. type of alkaline activator, source of aluminosilicates, etc.), leads to an increase in the compressive strength of the system^18^. Increasing the compressive strength of BA-WG-N (Si/Al molar ratio of 3.63) by 2 MPa compared to BA-N (Si/Al molar ratio of 3.24), as well as of BA-WG-RM10-N (Si/Al molar ratio of 5.98, which corresponds to the highest compressive strength obtained, namely 14.6 MPa) by 7.2 MPa compared to BA-N, clearly prove this trend. This significant increase in compressive strength could also highlight the positive effect of the addition of red mud to the synthesis mixes. This may be due to the reactive nature of its components, which could contribute to increasing the overall degree of geopolymerization of the system^19^. Red mud also could act as a filler with positive implications on the strength of the system^20^. The significant decrease in compressive strength for samples containing more than 10% red mud may be due to the excessive increase of the Si/Al molar ratio of the synthesis mixes (Table 2), which may affect the strength development both by direct effect on the gepolymerization process and indirectly through the so-called defect density^21^. Defect density derives from the unreacted fraction of the raw materials, which increases with increasing Si/Al molar ratio, and has a significant influence on the transformation and densification of the aluminosilicate gel derived from the reacted fraction of the raw materials during the geopolymerization process^22^.

Thereafter, only the geopolymer composites for which there has been a positive evolution of compressive strength, (i.e. BA-N, BA-WG-N, and BA-WG-RM10-N), will be characterized from mineralogical, morphological and structural point of view, and also tested for their leachability in relation to the contaminants contained.

### X-ray diffractometry

**Figure 2:**
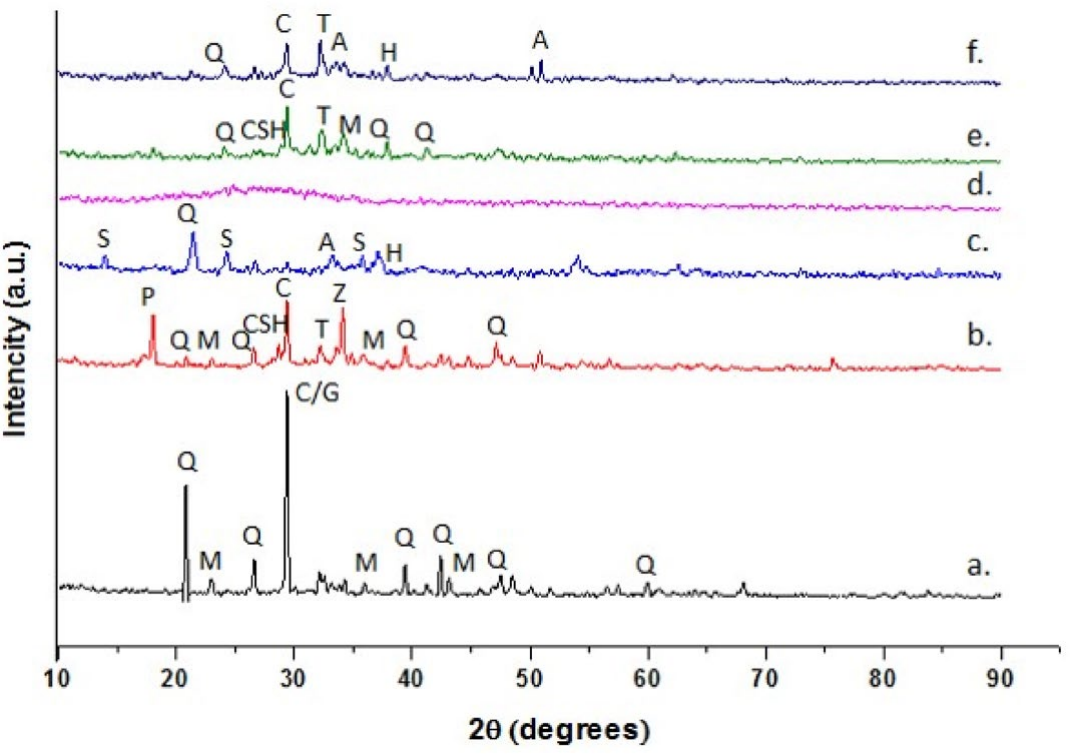
XRD patterns of aluminosilicate precursors and geopolymer composites: a. BA; b. BA-N; c. RM; d. WG; e. BA-WG-N; f. BA-WG-RM10-N. (Q – quartz , M – mullite, C – calcium carbonate, G – gypsum, T – thernadite, Z – zeolite, S – sodalite, P – portlandite, A – aluminate, H – hematite, CSH – calcium silicate hydrate).

XRD patterns of geopolymer precursors and synthesized geopolymer composites are shown in Fig. 2. As can be seen, there is no any broad hump in the BA pattern, which would be associated with its glassy content. This suggests a modest vitreous content, and a first sign of its low-reactivity. The main crystalline phases identified in BA are quartz (Q), mullite (M), calcite (C), and gypsum (G). The mineralogical composition of RM includes quartz (Q), sodalite (S), aluminate (A), and hematite (H). XRD pattern of WG shows a broad amorphous hump between 20 and 35° 2*θ*, which highlights its vitreous nature.

Comparative to the patterns of geopolymer precursors, those of the synthesized geopolymer composites present a series of differences, especially those for whose synthesis waste glass was used. In this respect, the broad amorphous hump centered at around 28° 2*θ* in WG pattern shifted to around 34° 2*θ*, in both BA-WG-N and BA-WG-RM10-N patterns, highlighting in this way structural changes that occurred during the alkaline activation of synthesis mixes. This shift could be associated with a significant change in the Si/Al and Na/Al molar ratios, which consequently influences the geopolymer microstructure^23,24^. Increasing the Si/Al molar ratio by adding waste glass, which in the alkaline environment of the geopolymeric system is an important source of dissolved silica, definitely influences the speciation of aluminum during the geopolymerization process (the higher the Si/Al molar ratio of the aluminosilicate solution, the lower the aluminum lability due to its incorporation in stable cyclic and larger aluminosilicate species), as well as the degree of its incorporation in the alkaline aluminosilicate gel, with consequences on its structure and conformation^22^. It should be noted that, after the alkaline activation, the peak corresponding to the gypsum phase in the BA pattern decreased considerably. This could be due to the reaction of gypsum with sodium hydroxide from the activation solution with the formation of new crystalline phases, namely thernadite (T) and portlandite (P). BA-N pattern confirms the presence of the T and P, and also shows the formation of two new mineral phases such as a zeolite phase (Z) and calcium silicate hydrate (C–S–H), the latter being also the characteristic of BA-WG-N pattern. It is worth mentioning that, because the activated synthesis mixes were sealed into plastic bags throughout their hardening, the portlandite (P) was not consumed in the reaction with the atmospheric carbon dioxide. This could be the reason why the characteristic peak of P is quite intense in the BA-N pattern. Contrary, the characteristic peak of P no longer appears in BA-WG-N and BA-WG-RM10-N patterns. This may be due to the consumption of P by reaction with the available silicon from the waste glass added to the synthesis mixture, leading to the formation of C–A–S–H gel^25^.

### Infrared spectroscopy

**Figure 3:**
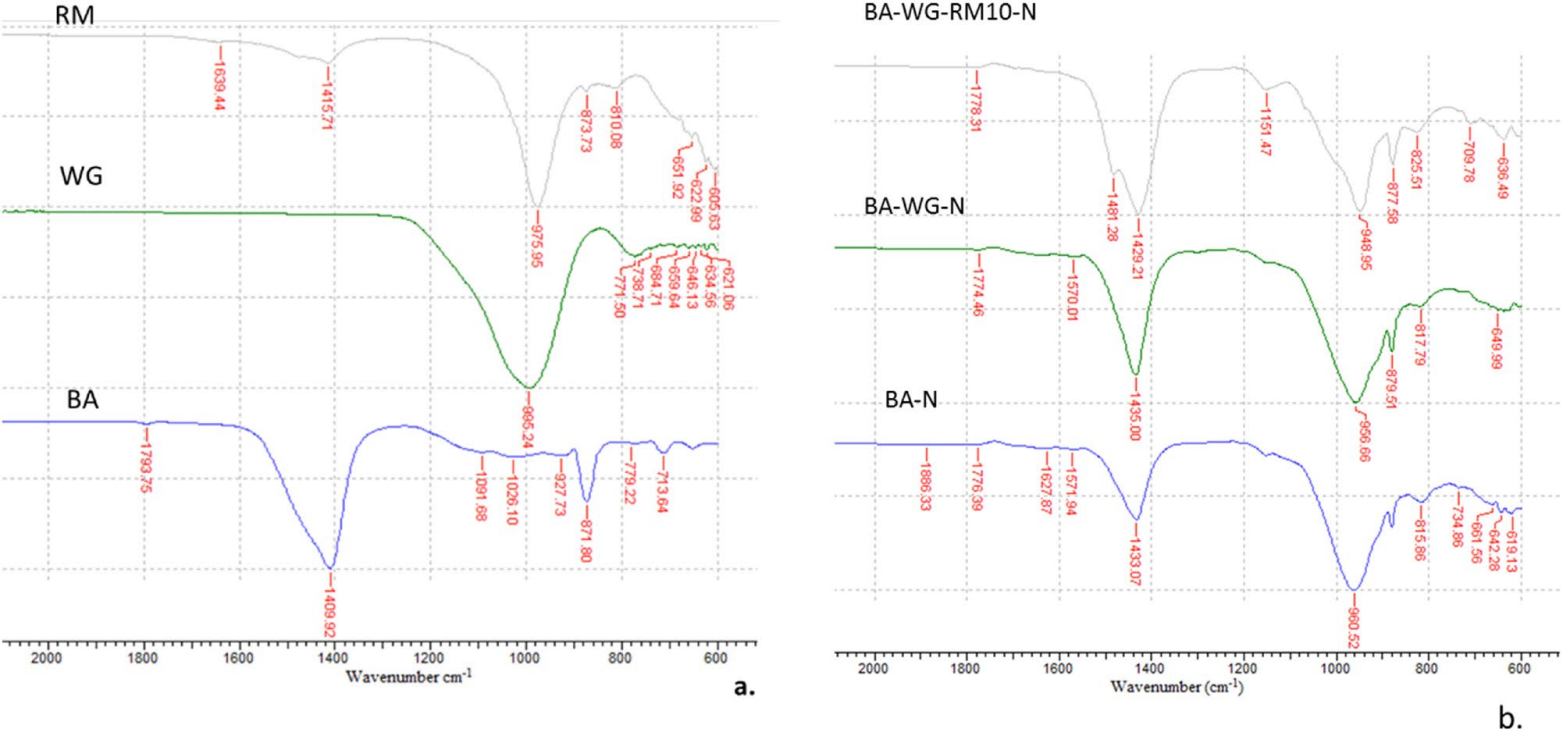
Infrared spectra for: a. aluminosilicate precursors, and b. geopolymer composites.

The FTIR spectra of both raw materials and geopolymer composites are presented in Fig. 3. As can be seen from Fig. 3b, the main absorption band in all the geopolymer composites is in the range of 949 to 961 cm^−1^, which is assigned to the asymmetric stretching vibration of the Si–O–T bonds (where T represents Si or Al tetrahedra) in aluminosilicates phases^26^. Originally, this absorption band is centered at about 1096 cm^−1^ in raw bottom ash, at about 995 cm^−1^ in raw red mud, and at about 976 cm^−1^ in raw waste glass (Fig. 3a). The left small absorption band at about 1151 cm^−1^ could be attributed to asymmetric stretching of T–O bonds that could be associated with the zeolite phase whose presence was highlighted by XRD analysis^27^. The absorption band in the range of 877 to 800 cm^−1^, as well as the absorption band in the range of 1429 to 1435 cm^−1^ corresponds to the stretching vibration of the carbonate groups O–C–O. This groups are associated with calcite (CaCO_3_) and thernadite (Na_2_CO_3_), whose presence was also highlighted by XRD analysis. The absorption bands in the frequency interval of 813–818 cm^−1^ are associated with the asymmetric stretching vibration of silanol groups Si–OH^28^. The absorption bands in the frequency interval of 619–633 cm^−1^ correspond to Si–O bending vibration of quartz.

The shifting of the Si–O–T band in the stretching mode is suggestive for the changes occurred in the framework of the geopolymer composites in regard with the Si/Al ratio. In this respect, a shift of this band toward a lower frequency (BA-N > BA-WG-N > BA-WG-RM10-N) denotes an increasing number of tetrahedral aluminum atoms in the framework of the geopolymer composites, which subsequently, leads to an increase in its number of three-dimensional cross-linked sites. The shift between BA-N and BA-WG-RM10-N is approximately 15 cm^−1^, which suggests that the addition of waste glass and red mud has led to significant changes in the microstructure of the geopolymer composites. In addition, the shifting of the Si–O–T band to a lower frequency in the geopolymer composites comparative with its position in the raw materials highlights the formation of alkaline aluminosilicate gel^26^.

### MAS-NMR spectroscopy

**Figure 4:**
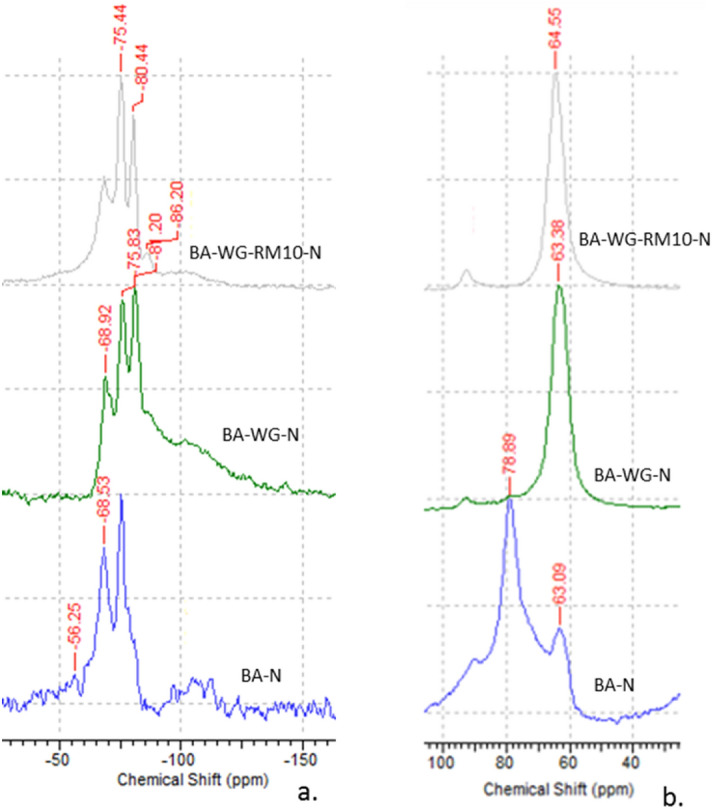
MAS-NMR spectra of geopolymer composites: (**a**) ^29^Si MAS-NMR spectra, (**b**) ^27^Al MAS-NMR spectra.

The ^29^Si and ^27^Al MAS NMR spectra for BA-N, BA-WG-N, and BA-WG-RM10-N geopolymer composites are shown in Fig. 4. The analysis of spectra was performed based on similar studies in this area^29–32^. As can be seen in Fig. 4a, the ^29^Si MAS NMR spectrum of BA-N exhibits one narrow resonance peak at -68.53 ppm which is related to monomeric silicates (Q_0_ units), and a resonance peak at − 75.44 ppm, which is attributed to the less condensed SiQ_1_(1Al) units. ^29^Si MAS NMR spectrum of BA-WG-N exhibits a resonance peak at − 75.83 ppm, which is associated with SiQ_1_(1Al) units, and a resonance peak at − 81.20 ppm which is attributed to SiQ_2_(1Al) and SiQ_2_(2Al) units. It should be also noted that the resonance peak that appears at around − 69 ppm related to Q_0_ units becomes much less intense in comparison with that of BA-N. ^29^Si MAS NMR spectrum of BA-WG-RM10-N exhibits a resonance peak at − 75.44 ppm, which is related to SiQ_1_(1Al) units, a resonance peak at − 80.44 ppm related to SiQ_2_(1Al) and SiQ_2_(2Al) units, and a small resonance peak at − 86.20 ppm, which is attributed to SiQ_4_(4Al) units. It is obvious that the geopolymer composites evolve, as new active sources of silicon and aluminum are added, from a weakly reacted composite with a structure in which the silicon is in isolated mono-groups (Q_0_ units), to a composite with an expanding three-dimensional cross-linked site (Q_4_ units).

^27^Al MAS NMR spectrum of BA-N (Fig. 4b) exhibits a sharp resonance peak at 78.89 ppm, which is attributed to the isolated aluminate groups (AlQ_0_), and a small resonance peak at 63.09 ppm, which is related to AlQ_3_(3Si) units (poly-sialate building units). ^27^Al MAS NMR spectra of BA-WG-N and BA-WG-RM10-N exhibit sharp resonance peaks at 63.38 and 64.55 ppm, both of them being associated with AlQ_3_(3Si) units. These results are consistent with those obtained through ^29^Si MAS NMR spectroscopy, and justify the results obtained from compressive strength tests.

### Scanning electron microscopy/energy-dispersive X-ray spectroscopy

**Figure 5:**
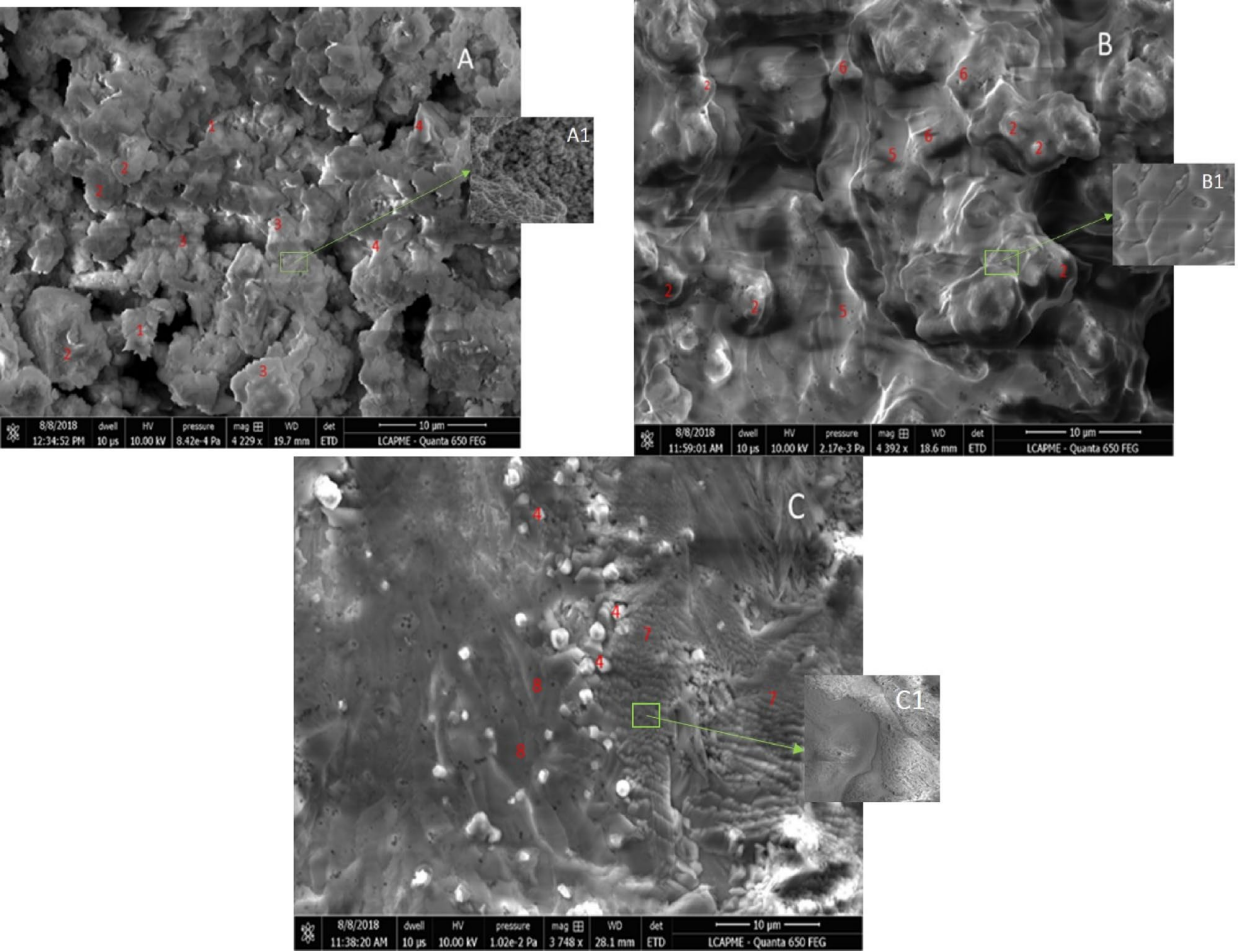
Scanning electron microscope images (4 kX; 10 μm) for: (**A**) BA-N, (**B**) BA-WG-N, and (**C**) BA-WG-RM10-N.

The micrograph of BA-N (Fig. 5A) exhibits unreacted bottom ash particles (1), bottom ash particles partially covered with the amorphous phase (2), amorphous phase matrix (3), as well as some crystalline particles (4), which are related to crystalline zeolites whose presence was highlighted by XRD analysis. The apparent continuity of the surface seems to be provided by the precipitation of some individual groups of particles that exhibit a good adhesion between them (Fig. 5A1). The micrograph of BA-WG-N (Fig. 5B) exhibits a continuous reaction mass (5) whose continuity seems to be provided by a solidified continuous gel that covers large portions of the precipitated material (Fig. 5B1). This may be due to the formation of a large amount Si-rich gel during the activation of the waste glass powder. The micrograph also revealed ash particles covered with the amorphous gel (2), as well as a series of smooth surfaces (6) associated with the unreacted waste glass particles that are also covered with amorphous gel. The micrograph of FA-WG-RM10-N (Fig. 5C) exhibits a series of crystalline particles (4), which are related to crystalline zeolites and large areas covered with amorphous phase (7). In addition to the areas related to amorphous phase (Fig. 5C1), there are other areas that look different, probably located next to the unreacted or partially reacted glass particles that are attributed to Si-rich areas (8).

**Figure 6:**
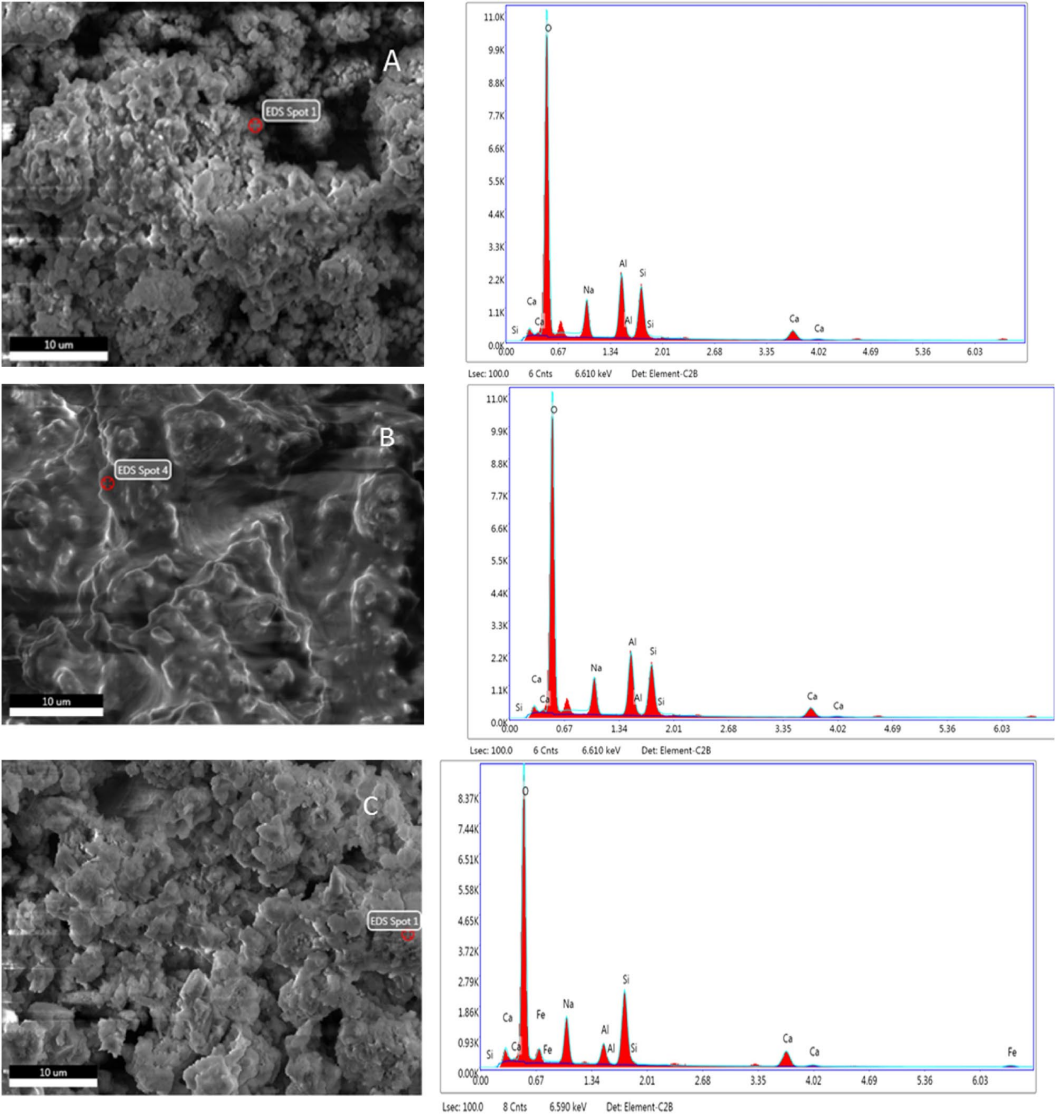
EDX spectra for: (**A**) BA-N; (**B**) BA-WG-N, and (**C**) BA-WG-RM10-N.

The EDX spectra for the selected areas where the amorphous phase is assumed to exist within BA-N, BA-WG-N, and BA-WG-RM10-N SEM images (Fig. 6) indicate a Si/Al molar ratio of 2.10 for BA-N, 2.21 for BA-WG-N, and 1.92 for BA-WG-RM10-N, as well as a Na/Al molar ratio of 1.71 for BA-N, 1.52 for BA-WG-N, and 1.32 for BA-WG-RM10-N. The values of these molar ratios indicate the formation of N–A–S–H gel, but at the same time, the value of Ca/Si molar ratio ranging from 0.42 to 0.50 suggests the presence of a low calcium C–S–H gel, whose presence was also revealed by XRD analysis. Although there is not enough evidence, C–A–S–H or (N, C)–A–S–H gels could also coexist in the system, under certain conditions, with the gels mentioned above. A pozzolanic reaction between portlandite, which was confirmed for BA-N by XRD analysis (Fig. 2), and available silicon derived from waste glass could lead to the formation of a C–A–S–H type gel^25^. In addition, the Ca/Si molar ratio established by EDX analysis is lower than its normal value for C–S–H gel which is in the range of 0.67 to 1.5^33^. This may be due to the substitution of some Si with Al to form Al-substituted C–S–H gel or C–A–S–H type gel^24^.

It is worth noting that the EDX analysis shows that the Na/Al molar ratio decreases in order of BA-N > BA-WG-N > BA-WG-RM10-N. The optimum theoretical Na/Si molar ratio required for charge balance in the bonding network is 1.00. Therefore, an excessive increase in this ratio leads to a significant decrease in the mechanical strength of the resulting geopolymers^34^. This may be another reason why compressive strength of the geopolymer composites increases in order BA-N < BA-WG-N < BA-WG-RM10-N.

### Mössbauer spectroscopy

**Figure 7:**
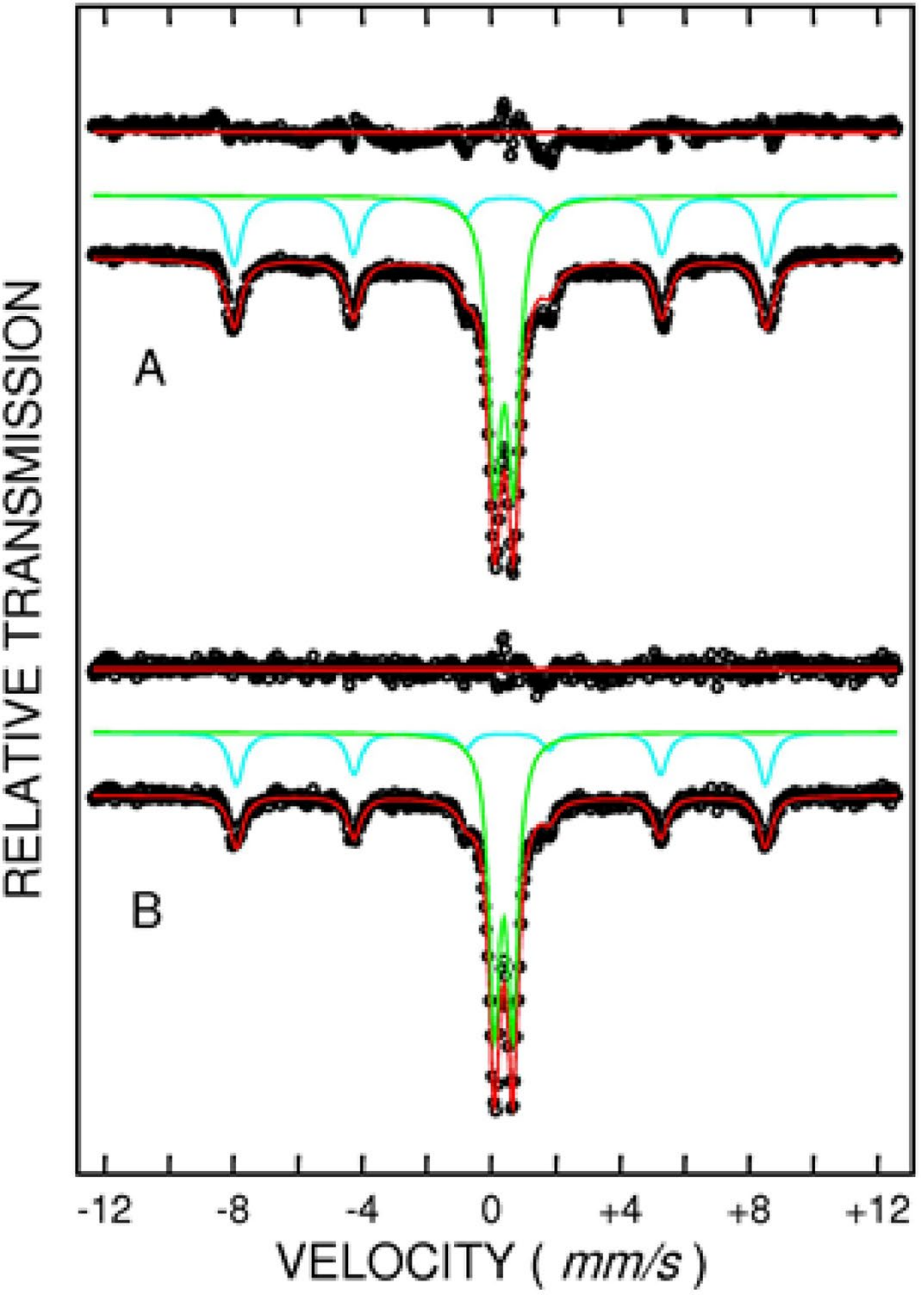
Mössbauer spectra at 300 K for: (**A**) RM and (**B**) BA-WG-RM10-N.

Mössbauer spectra measured at ambient temperature for RM and BA-WG-RM10-N are shown in Fig. 7. The hyperfine parameters extracted from Mössbauer fit are: (i) Isomer shift (IS), which is proportional to the electron density to iron nucleus and gives information about valence state and coordination, (ii) Quadrupole splitting (QS), which is proportional to the electric field gradient to iron nucleus and provides information about valence state and local distortion, (iii) Hyperfine magnetic field (HF), which characterizes the magnetic phases. The hyperfine parameters obtained by fitting the Mössbauer spectra from Fig. 7A, B are given in Table 3.

**Table 3:** Mössbauer hyperfine parameters for RM and BA-WG-RM10-N.

Sample	Component	IS (mm/s)^a^	QS (mm/s)	HF (T)	Line width (mm/s)	Relative area (%)
RM	Sextet	0.39(1)	0.23(1)	51.20(3)	0.53	40.2
	Doublet	0.40(1)	0.59(1)	–	0.47	59.8
BA-WG-RM	Sextet	0.39(1)	0.20(1)	50.90(3)	0.51	33.6
	Doublet	0.38(1)	0.57(1)	–	0.40	66.4

^a^IS values are referred to metallic iron.

The Mössbauer spectra were well fitted with a sextet corresponding to hematite (α-Fe_2_O_3_) and a central doublet corresponding to a compound which contains Fe^3+^. The hyperfine field corresponding to hematite in RM (51.20 T) is very close to the value 51.15 T given in literature at 300 K^35^. In BA-WG-RM10-N the hyperfine field of hematite is 50.90 T with very little less than that reported in literature. This suggests that hematite from BA-WG-RM10-N contains some impurity atoms (Al, Si, etc.). The central doublet from RM sample has IS = 0.40 mm/s and QS = 0.59 mm/s which can be assign to Fe^3+^ with octahedral coordination in silicates or aluminosilicates. Similar values were reported in literature for andradite garnet silicates^36^, sol–gel iron-containing silicate glasses^37^, iron-containing aluminosilicate glasses^38^, and chemical treated kaolin^39^.

In BA-WG-RM10-N the hyperfine parameters of the central doublet are IS = 0.38 mm/s and QS = 0.57 mm/s which are very close to that found for RM. The relative area of each spectral component gives information about the relative amount of each iron-containing phase. The ratio R = (iron belonging to central doublet)/(iron contained in hematite) was estimated for the two samples subjected to analysis, namely R = 1.49 for RM and R = 1.98 for BA-WG-RM10-N, which means a significant increase of the doublet (Fe^3+^ with Si and Al environment) in BA-WG-RM10-N compared with the original RM. Consequently, Fe^3+^ with octahedral coordination in iron-containing aluminosilicate phase is significantly higher in BA-WG-RM10-N compared with RM. This might suggest the formation of some new sixfold-coordinated ferric sites most probably located at amorphous gel-like phases^40^. These amorphous iron-rich phases have different structural characteristics than the typical aluminosilicate structure of the geopolymer gel, and certainly the presence of iron into the adhesive bonds influences the overall strength of the geopolymer composite^41^.

### Leachability

XRF analysis (Table 4) revealed that the raw materials contain, to a greater or lesser extent, contaminants that under certain conditions could leach into environment in which the obtained geopolymer composites could arrive.

**Table 4:** Initial content of contaminants in raw materials.

Raw material	Contaminant, % (by weight)					
	PbO	BaO	CuO	ZnO	NiO	Cr_2_O_3_
Bottom ash	0.05	0.12	0.10	0.08	0.01	0.04
Waste glass	12.10	6.97	0.04	0.12	0.02	0.01
Red mud	0.01	0.04	0.01	0.13	0.06	0.25

Therefore, the testing of the leaching behavior is an important step in the process of evaluating the performance of the obtained materials. In this work, the leaching behavior was tested by using a semi-dynamic tank leaching procedure and the obtained results were used to assess the leaching rates for target contaminants through the leachability indexes (LI) of the monolithic samples^15^. It is worth mentioning that the increasing of LI denotes a decreasing of leaching rate of the contaminants. The leaching test results are shown in Table 5.

**Table 5:** Tank leaching test results for geopolymer composites.

Geopolymer composite	Leachability index (LI)					
	Pb	Cu	Zn	Ni	Cr	Ba
BA-N	13.4	13.1	12.8	13.8	12.6	12.7
BA-WG-N	12.2	13.0	13.4	13.5	13.0	12.4
BA-WG-RM10-N	12.6	13.5	13.8	14.1	13.0	12.9

As can be seen, LI values highlight a low or moderate mobility of contaminants in the geopolymer composites. Only Pb and Ba, which are present in a significant amount in the waste glass, exhibit an average mobility in BA-WG-N, but their mobility in BA-WG-RM10-N becomes low. In fact, the general tendency is that LI of all contaminants increases in BA-WG-RM10-N, which proves a better capacity to immobilize the contaminants of this type of geopolymer composite. If it is assumed that the main immobilization mechanism is physical entrapping of contaminants into the material network, then these results could be correlated with those obtained from compressive strength tests. Therefore, the higher the strength of the matrix, the lower the mobility of contaminants. However, additional research has to be done in order to establish the immobilization mechanisms involved.

## Conclusions

The influence of adding alternative geopolymeric precursors such as waste glass and red mud on the microstructural, mechanical, and leaching properties of bottom ash-based geopolymer was studied in this work. The results revealed that adding up to 10% red mud to the synthesis mixes leads to an increase in the degree of geopolymerization of the activated mixes. In this respect, the spectroscopic analysis highlighted that the geopolymer composites evolve from a weakly reacted composite to a composite with an expanding three-dimensional cross-linked site. Mössbauer spectroscopy revealed that, Fe^3+^ with octahedral coordination in iron-containing aluminosilicate phases is significantly higher in geopolymer composites containing red mud compared with the original red mud. This might suggest the formation of some new ferric sites located at amorphous gel-like phases in which the Fe^3+^ is in six-fold-coordination.

The best results in terms of compressive strength were obtained for the geopolymer composites synthesized by adding waste glass and red mud in amount of 10% by each to synthesis mixes. A further increase in the amount of red mud added to the synthesis mixes leads to a significant decrease in compressive strength.

The leaching test results showed a low mobility of the contaminants especially in the geopolymer composites for which good results have been obtained regarding the compressive strength.
